# Applications of artificial intelligence and machine learning in orthodontics: a scoping review

**DOI:** 10.1186/s40510-021-00361-9

**Published:** 2021-07-05

**Authors:** Yashodhan M. Bichu, Ismaeel Hansa, Aditi Y. Bichu, Pratik Premjani, Carlos Flores-Mir, Nikhilesh R. Vaid

**Affiliations:** 1 Abu Dhabi, United Arab Emirates; 2 Durban, South Africa; 3 Dubai, United Arab Emirates; 4 Department of Orthodontics, University of Alberta, Edmonton, Alberta Canada; 5 Department of Orthodontics, European University College, Dubai, United Arab Emirates

**Keywords:** Artificial intelligence, Machine learning, Orthodontics

## Abstract

**Introduction:**

This scoping review aims to provide an overview of the existing evidence on the use of artificial intelligence (AI) and machine learning (ML) in orthodontics, its translation into clinical practice, and what limitations do exist that have precluded their envisioned application.

**Methods:**

A scoping review of the literature was carried out following the PRISMA-ScR guidelines. PubMed was searched until July 2020.

**Results:**

Sixty-two articles fulfilled the inclusion criteria. A total of 43 out of the 62 studies (69.35%) were published this last decade. The majority of these studies were from the USA (11), followed by South Korea (9) and China (7). The number of studies published in non-orthodontic journals (36) was more extensive than in orthodontic journals (26). Artificial Neural Networks (ANNs) were found to be the most commonly utilized AI/ML algorithm (13 studies), followed by Convolutional Neural Networks (CNNs), Support Vector Machine (SVM) (9 studies each), and regression (8 studies). The most commonly studied domains were diagnosis and treatment planning—either broad-based or specific (33), automated anatomic landmark detection and/or analyses (19), assessment of growth and development (4), and evaluation of treatment outcomes (2). The different characteristics and distribution of these studies have been displayed and elucidated upon therein.

**Conclusion:**

This scoping review suggests that there has been an exponential increase in the number of studies involving various orthodontic applications of AI and ML. The most commonly studied domains were diagnosis and treatment planning, automated anatomic landmark detection and/or analyses, and growth and development assessment.

**Supplementary Information:**

The online version contains supplementary material available at 10.1186/s40510-021-00361-9.

## Introduction

The use of information technology (IT) in the dental field has increased significantly over the past 25 years and has helped reduce cost, time, dependence on human expertise, and medical errors. As a subfield of computer science, artificial intelligence (AI) encompasses both hardware and software that can perceive its environment and take action that maximizes its chances of successfully achieving its goals [1–4]. Although developments in AI had started in 1943 [5], it was only in 1956 that the term was coined by John McCarthy and adopted during a meeting at Dartmouth College [6]. AI allows examination, organization, representation, and cataloging of medical information, and its robust pattern finding and prediction algorithms are helping drive discoveries across all sciences [7]. In 2019, Morgan Stanley estimated that the global market for AI in healthcare could surge from $1.3 billion to $10 billion by 2024, growing at an annual compound rate of 40% [8].

While AI is a broad term and includes various classifications, there are two main categories of AI: symbolic AI and machine learning from an algorithmic perspective. Symbolic AI is a collection of techniques based on structuring the algorithm in a human-readable symbolic manner. This category was the paradigm of AI research until the late 1980s and is widely known as GOFAI—good old-fashioned AI [9]. AI is still used for solving problems in which the possible outcomes are limited, computational power is scarce, or human explainability is essential. However, in healthcare, where problems tend to be complex, not always fully understood, and have many explanatory variables, building a model based on a limited set of rules is extremely difficult, if not impossible [10].

Machine learning (ML)—a term first phrased by Arthur Samuel in 1952, is the current paradigm. The fundamental difference between ML and symbolic AI is that, in ML, the models learn from examples rather than a set of rules established by a human [7]. By utilizing a mixture of statistical and probabilistic tools, machines can learn from previous models and improve their actions when new data is introduced. This could be in the form of predictions, identifying new patterns, or classifying new data. ML can be categorized into three types, depending on the type of learning of the algorithm and the chosen outcome: supervised learning (used for classification or prediction based on a known outcome), unsupervised learning (finding hidden patterns and structures with unknown outcomes), and reinforcement learning (machine develops a modified algorithm based on previous versions that maximizes the intended reward) [11].

Deep learning (DL) is a sub-domain of ML in which the machine itself calculates specific features of a given input. DL’s precursor is an artificial neural network (ANN), which was initially developed in the 1900s. With the exponential increase in computational technology and power, researchers have designed more complicated and “deeper” neural networks to solve more complicated practical problems. The neural network has become known as “deep learning.” [12].

Algorithms used for ML may also be used for data mining. Data mining applies these algorithms to historical data to identify new relationships or patterns and therefore aid practitioners in optimizing decision-making in their daily practice, as well as improving quality of care [13, 14]. Alternatively, if predictions are desired, ML should be utilized. For example, a practitioner can use existing data about a disease to train the machine to calculate predictions about the diagnosis or prognosis of patients that has not yet been seen before. Notably, ML predictive models have been shown to have greater accuracy than statistic-based models [15].

In recent years, scoping reviews have become an increasingly adopted approach and have been published across various social sciences and healthcare fields. Orthodontics has been a slow starter on this terrain [16]. Scoping reviews are of particular use when a body of literature has not yet been comprehensively reviewed or exhibits a large, complex, or heterogeneous nature not amenable to a more thorough systematic review [17].

Current orthodontic literature is replete with studies that have documented various applications of AI and ML, utilizing different types of algorithms mentioned above, albeit in isolation. No study hitherto, has attempted to systematically organize the existing literature to review existing AI and ML applications in orthodontics, classify the types of algorithms applied, and provide a comprehensive mapping of studies conducted in this field. Hence, this scoping review aims to provide an overview of the existing evidence of how far the earlier AI and ML advancements in orthodontics have translated into clinical fruition and the limitations that have precluded their envisioned development. The authors have attempted (1) to chart the evolution of AI in the orthodontic field over the years, (2) to examine the utilization of applications of AI and ML in the field of orthodontics, and (3) to collate the type of artificial intelligence algorithms that have been implemented in orthodontics.

## Materials and method

A scoping review of the published literature was performed following the Preferred Reporting Items for Systematic Reviews and Meta-Analyses extension for Scoping Reviews (PRISMA-ScR) guidelines. This protocol was not registered previously.

The eligibility criteria of the scoping review are outlined in Table 1.

**Table 1:** Scoping review eligibility criteria

Inclusion criteria	Exclusion criteria
1. Randomized controlled trials (RCTs), prospective or retrospective cohort studies.	1. Case-control studies, cross-sectional studies, case reports or case series.
2. Any type of comparison between AI- or ML-based method and conventional mode of orthodontic treatment, method or approach.	2. Personal opinion, descriptive paper, letter to editor or interviews.
3. All types of reported outcomes (primary and secondary) related to AI or ML outputs.	3. Technique articles (that focus on design description).
	4. Proof of concept.

The first author (YMB) conducted an initial literature search in PubMed on 2 July 2020 with the keywords listed in Table 2.

**Table 2:** Keywords for initial literature search

1.	‘artificial intelligence and orthodontics’
2.	‘machine learning and orthodontics’
3.	‘deep learning and orthodontics’
4.	‘automatic detection algorithms and orthodontics’
5.	‘neural networks and orthodontics’
6.	‘hybrid approach and orthodontics’

No restrictions were made concerning year or publication status; however, studies with missing English abstracts were excluded. After eliminating duplicate studies, three of the authors (YMB, AYB, NRV) independently screened the titles and abstracts of the retrieved citations to exclude non-eligible articles based on the study’s eligibility criteria and keywords. A copy of the full text was obtained for the articles considered potentially useful after this selection stage. The same authors then read each full-text article to determine whether it met the inclusion criteria. Additional material about the articles included as an appendix was acquired when needed, and the reference lists of acquired articles were also searched for relevant articles. Any disagreement was resolved by discussion between three authors (YMB, AYB, NRV) until a final consensus was achieved.

### Data extraction

Data extraction was charted according to “PICO” guidelines and can be found in the supplementary materials. The collected information included the author names, year of publication, country of origin, whether the study was published in an orthodontic specialty journal, study population or sample size, intervention type, comparator, the outcome of the intervention, type of AI algorithm employed, and finally a broad-based orthodontic outcome domain.

## Results

**Fig. 1:**
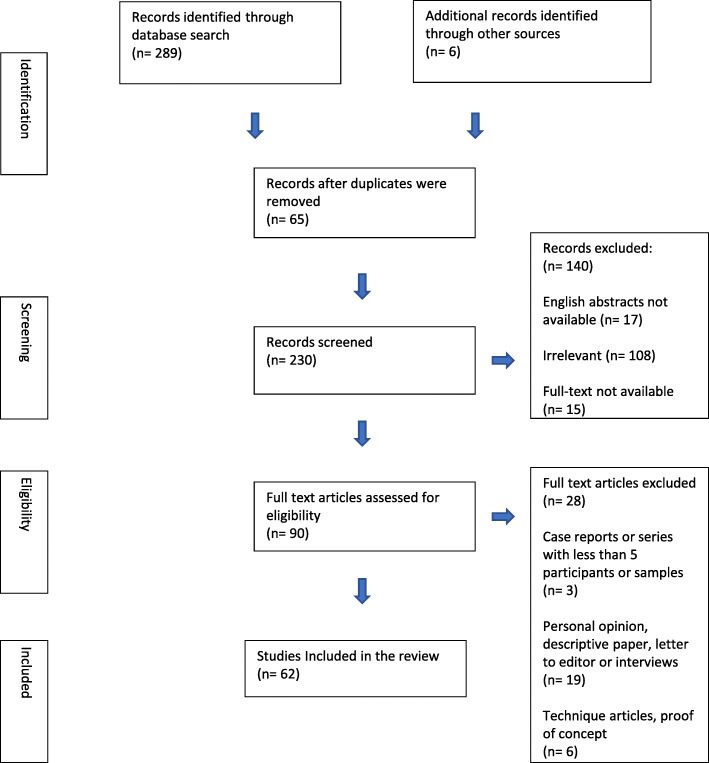
PRISMA flow diagram of the scoping review

The initial search strategy resulted in 289 records, 65 of which were excluded as duplicates, 6 studies that did not appear in the initial search strategy but were identified through references were added subsequently, resulting in 230 records screened for further eligibility. A total of 140 records were excluded as they were irrelevant to the topic of AI/ML applications in orthodontics (n = 108), did not feature an English abstract (n = 17), and finally because the full-text articles of these records could not be retrieved (n = 15). Hence, 90 full-text articles were then evaluated for eligibility, of which case series or case reports (n = 3), personal opinions/descriptive papers/interviews (n = 19), and technique articles/proof of concept (n = 6) were excluded, resulting in 62 articles that fulfilled the inclusion criteria of this study (Fig. 1 illustrates the PRISMA-ScR flowchart for the scoping review). The details of the studies included in the scoping review are listed in Supplementary table 1.

In regard to the date of publication, 43 out of the 62 studies (69.35%) included in this scoping review were published between 2011 and 2020, 12 studies (19.35%) between 2001 and 2010 and 7 studies (11.3%) were published between 1991 and 2000 (Supplementary table 2). Results reveal that 11 studies originated from the USA, 9 from South Korea, 7 from China, 6 each from Japan and Italy, 4 from Turkey, 3 each from Brazil, India, Germany, and the UK, 2 from Switzerland, Mexico, Colombia, Spain, and 1 study each from Russia, Morocco, Thailand, Iran, Serbia, Singapore, and Australia (Supplementary table 3). Thirty-six out of the 62 studies were published in non-orthodontic journals (58%) whereas 42% (26 studies) were published in orthodontic specialty journals (Supplementary table 4).

Artificial neural networks (ANNs) were found to be utilized as the AI/ML algorithm in 13 studies, convolutional neural networks (CNNs) and support vector machine (SVM) in 9 studies and regression in 8 studies apart from 23 other algorithms utilized in various studies. Results classified as per the type of AI algorithm employed in the study are listed in Table 3.

**Table 3:** Studies classified by AI/ML algorithm employed

Type of AI/ML algorithm utilized	Number of studies	Reference number
Artificial neural network—ANN	13	[18–30]
Convolutional neural network—CNN	9	[31–39]
Support vector machine	9	[20, 24, 40–46]
Regression	8	[18, 20, 33, 39, 42, 47–49]
Random forest	5	[18, 20, 47, 50, 51]
Decision tree	3	[20, 41, 50]
Bayesian networks	3	[52–54]
Expert systems	3	[55–57]
Active shape models	2	[58, 59]
Automatic detection algorithms	2	[60, 61]
Fuzzy clustering	2	[62, 63]
Active appearance models	2	[64, 65]
Shape variation analyzer	2	[66, 67]
Pattern matching	2	[68, 69]
Light GBM	1	[47]
XG boost	1	[47]
Deep learning network	1	[70]
k-nearest neighbors	1	[20]
Naïve-Bayes	1	[20]
Machine learning with LINKS	1	[71]
Gaussian process regression—GPR	1	[40]
Logic learning machine	1	[72]
Mean shift algorithm	1	[43]
Network analysis	1	[73]
Projected principle edge distribution	1	[74]
Spatial spectroscopy	1	[75]
Unspecified algorithms	4	[76–79]

The study results also helped classify artificial intelligence applications into 4 core domains and a 5th domain that clubbed together miscellaneous applications. Table 4 enlists results classified as per domains of applications of artificial intelligence in orthodontics.

**Table 4:** Studies classified as per domains of applications of artificial intelligence in orthodontics

The domain of application of artificial intelligence in orthodontics	Number of studies	Reference number
Diagnosis and treatment planning:		
a. For orthodontic extractions	5	[18, 23, 25, 28, 68]
b. For TMJ Osteoarthritis	4	[22, 47, 66, 67]
c. To assess maxillary constriction and/or impacted canines	3	[51, 54, 71]
d. For screening of osteoporosis from panoramic radiographs	2	[34, 41]
e. Assessment for need for orthodontic treatment and/or prediction of treatment outcome	2	[42, 53]
f. Classification of skeletal patterns	2	[24, 44]
g. Prediction of orthodontic treatment outcome—class III M/O	2	[46, 62]
h. For orthognathic surgery and orthodontic extractions	1	[21]
i. To assess airflow dynamics, predict upper airway collapsible sites and obstructive sleep apnea	1	[40]]
j. To predict association between *C. difficile* infections in hospitalized patients with major surgeries	1	[52]
k. Genetic risk assessment for non-syndromic orofacial cleft	1	[48]
l. To predict occurrence of obstructive sleep apnea in patients with Down’s syndrome	1	[72]
m. Evaluation of facial attractiveness	1	[45]
n. Trainers for clenching	1	[29]
o. Selection of orthodontic appliance- type of headgear	1	[63]
p. Quantification of sagittal skeletal discrepancy	1	[49]
q. For cases suitable for fixed mechanotherapy	1	[55]
r. Selection of patients suitable to be treated with removable orthodontic appliances	1	[56]
s. Class II division 1 malocclusion	1	[57]
t. Broad-based	1	[79]
Automated cephalometric landmarking and/or analysis and/or classification		
a. Lateral cephalogram	12	[27, 32, 35, 37, 64, 65, 69, 70, 74, 75, 77, 78]
b. CBCT images	6	[31, 58–61, 76]
c. Frontal cephalogram	1	[19]
Assessment of growth and development		
a. Cervical vertebra maturation	1	[18]
b. Broad-based	3	[30, 39, 73]
Evaluation of treatment outcome- orthognathic surgery on facial appearance/ attractiveness and/or age perception	2	[36, 38]
Miscellaneous		
a. Tooth segmentation from CBCT images/model	2	[33, 43]
b. Detection of activation pattern of tongue musculature	1	[50]
c. Evaluation of temperature changes during curing for orthodontic bonding	1	[26]

## Discussion

There has been a steady increase in the number of scoping reviews published in orthodontic literature over the last few years [80, 81]. Through this scoping review, the authors have attempted to organize the existing literature in a systematic manner in order to document existing applications of AI and ML in the field of orthodontics.

The study results reveal that one of the earliest studies to document the utilization of AI in the field of orthodontics was published in 1986 in a non-orthodontic journal; however, this was excluded from this scoping review based on its inclusion criteria [82]. The first study included in this review was found to be published in 1991 [57]; between 1991 and 2000, 2001 and 2010, and 2011 and 2020, there was a progressive increase in publications from 7 to 12 and 43 respectively, clearly indicating an exponential rise in this subject due to technological advancements and the continuous digitization of orthodontics.

These studies were primarily published in non-orthodontic journals (36) compared to orthodontic specialty journals (26). This is perhaps reflective of the far-reaching applications of AI and ML and points out toward possible scope of a further collaboration of various disciplines in the field of AI and ML.

This distribution map of research undertaken in the field of AI in orthodontics shows that the majority of the studies originated from the USA (11), South Korea (7), and Japan and China (7 each). Apart from these 4 countries, studies were also found to originate from 17 other countries, reflecting the more considerable increase in AI and ML’s interest and their subsequent utilization in orthodontics.

This scoping review also tried to examine the types of AI algorithms commonly employed in various studies. The results reveal that artificial neural networks (ANNs) were the widely utilized AI/ML algorithm (10) followed by convolutional neural networks (CNNs), support vector machine (SVM)-8 studies and regression (logistic and linear) in 8 studies apart from 23 other algorithms utilized in various studies.

The research question—“what are AI and ML applications in the field of orthodontics?” threw up five major domains. Each domain was addressed with the PICO framework for literature evaluation and can be enumerated as (1) diagnosis and treatment planning—either broad based or specific, (2) automated anatomic landmark detection and/or analyses, (3) assessment of growth and development, (4) evaluation of treatment outcome and finally a (5) miscellaneous category.

Our study results show that the maximum number of publications focused on automated anatomic landmark detection and/or analyses as the major domain of AI utilization, chiefly from lateral cephalograms, more recently from CBCT images and lastly from frontal cephalograms. This review’s first study to utilize AI for automatic extraction of cephalometric landmarks was published back in 1998 in an orthodontic specialty journal [75]. Several studies since then [27, 32, 35, 37, 64, 65, 69, 70, 74, 77, 78] have affirmed greater accuracy of landmark detection, reduced time, and human effort spent on anatomic landmark detection and/or analyses with AI/ML as compared to traditional methods.

Studies have shown that cephalometric analysis’s angles and lengths, predicted by neural networks, were not statistically significant from those calculated from manually plotted points. Yu et al. [32] proposed a system that exhibited > 90% sensitivity, specificity, and accuracy for vertical and sagittal skeletal diagnosis and concluded that CNN-incorporated system showed potential for skeletal orthodontic diagnosis without the need for intermediary steps requiring complicated diagnostic procedures. The use of CBCT for cephalometric analysis has now become commonplace. Various studies [31, 58–61, 76] that have employed AI and ML techniques for automatic landmark detection and analysis have shown that the results obtained are as accurate and less time-consuming as compared to those obtained with manual analysis. At least one study [19] included in this review compared frontal cephalometric landmarking ability of humans versus that of artificial neural networks and the results showed that ANNs could achieve accuracy comparable to humans in placing cephalometric points, and in some cases surpasses the accuracy of inexperienced doctors (students, residents, graduate students).

The second domain incorporating AI and ML utilization was broadly labeled as diagnosis and treatment planning, with applications intended either for broad-based or specific clinical situations. Diagnosis remains the cornerstone of successful orthodontic treatment. However, thus far, no tools exist to lead patients and clinicians out of the decision-making uncertainty in which they are trapped, especially when they face a condition that has several possible correct treatment options and orthodontists over the years have attempted to create systems that take the subjective bias out of diagnostic decision-making.

Expert systems [55–57, 79] are one of the earliest and most basic implementations of AI and have been popular for diagnosis and treatment planning in the medical and dental fields. They process the input information and provide solutions based on “if-then” rules. “If-then” based expert systems are limited to currently existing data when the system is created, and regular updates are required to ensure that the outcomes is correct and up to date. Rule-based expert systems have now become obsolete due the aforementioned limitations, and the development of newer technologies such as ML.

To “extract or not to extract” has been the question in orthodontics since time immemorial, with substantial variability noted between orthodontists’ decisions. Unsurprisingly, the most significant number of studies [18, 23, 25, 28, 68] under the diagnosis domain was found to investigate the development of several decision support systems that reduce the relative subjectivity or increased complexity of making the extraction decision. Artificial neural networks (ANNs) were used to develop such systems, and they were shown to be successful at predicting the extraction decision with an accuracy of 94% for extraction decision, 84.2% for extraction pattern determination and 92.8% for anchorage pattern determination [23]. This study suggested that ANNs could provide good guidance for orthodontic treatment planning for less experienced orthodontists. Choi et al. [21] expanded the use of ANNs to determine the diagnosis of orthognathic surgery in addition to the extraction decision and their study results showed a 96% success rate for diagnosis of surgery/non-surgery decision and 91% success rate for detailed diagnosis of surgery type and extraction decision. A recent study [18], when comparing all classifiers, concluded that random forest classifier outperforms neural network model for the prediction of the specific extraction treatment.

Four studies included in this scoping review were dedicated toward the assessment of need for orthodontic treatment and/or prediction of treatment outcome [42, 46, 53, 61]. Thanathornwong [53] utilized the Bayesian network (BN) for assessment of the need for orthodontic treatment and concluded that the results obtained by the decision support system were comparable with those suggested by expert orthodontists. Wang et al. [42] explored the function of an eye-tracking method to evaluate orthodontic treatment need and treatment outcome from the lay perspective in an objective way when compared to traditional methods. The authors employed support vector machine techniques and concluded that the eye-tracking device was able to objectively quantify the effect of malocclusion on facial perception and the impact of orthodontic treatment on malocclusion from a lay perspective.

Predictions of treatment outcomes in class II and class III patients have also been reported. Auconi et al. developed a system to predict outcomes in untreated class III patients [62]. Unsupervised learning was used to cluster patients as hypermandibular, hyperdivergent, or balanced based on cephalometric variables. The system was then applied to a treated sample, where it showed that all of the unsuccessful cases belonged to either the hypermandibular or the hyperdivergent cluster. The same author [73] also attempted to identify critical peculiarities of class II and class III malocclusions and demonstrated that class II subjects exhibited few highly connected orthodontic features, while class III patients showed more compact network structure characterized by strong co-occurrence of normal and abnormal clinical functional and radiological features. The study concluded network analysis could allow orthodontists to visually evaluate and anticipate the co-occurrence of auxological anomalies during individual craniofacial growth and possibly localize reactive sites for a therapeutic approach to a malocclusion.

A group of researchers have specifically studied the applications of AI and ML for the detection of TMJ osteoarthritis [22, 47, 66, 67] and have concluded that deep learning neural network was the most accurate method for classification of TMJ-OA that allows disease staging of bony changes in TMJ-OA. The authors expected their efforts to boost future studies into early detection and osteoarthritis patient-specific therapeutic interventions, and thereby improve the health of articular joints.

Three studies in this scoping review focused on the assessment of maxillary constriction and/or maxillary canine impactions [51, 54, 71]. Chen et al. [71] developed a machine learning algorithm utilizing Learning-based multi-source IntegratioN frameworK for segmentation (LINKS) used with CBCT images to quantify volumetric skeletal maxilla discrepancies and suggested palatal expansion could be beneficial for those with unilateral canine impaction, as underdevelopment of maxilla often accompanies canine impaction in early teen years. Another study [51] concluded that among learning machine methods tested to classify data, the best performance was obtained by random forest method, with an overall accuracy of 88.3% in predicting canine eruption. The authors performed measurements on 2D routinely executed radiographic images, found them to be independently related to canine impaction and showed reliable accuracy in predicting maxillary canine eruption. Bayesian network analysis [54] showed bilateral impaction was associated with palatal impactions and longer treatments, pre-treatment alpha-angle was a determinant for the duration of the orthodontic traction and the post-treatment periodontal outcome was not related to pretreatment radiographic variables.

One of the challenges for less experienced orthodontists is the selection of the appropriate treatment modality and appliance. A system was developed to help orthodontists select the appropriate type of headgears [63]. Compared to the selections made by eight expert orthodontists, the system correctly identified the appropriate headgears 95.6% of the time.

Isolated studies included in this scoping review under the domain of diagnosis and treatment planning have also investigated the applications of AI and ML for screening of osteoporosis from panoramic radiographs [34, 41]; assessment of airflow dynamics, prediction of upper airway collapsible sites, and obstructive sleep apnea [40]; prediction of the association between *C. difficile* infections in hospitalized patients with major surgeries [52]; genetic risk assessment for non-syndromic orofacial cleft patients [48] and for prediction of occurrence of obstructive sleep apnea in patients with Down’s syndrome [72].

The third domain of applicability of AI can be described as the evaluation of orthodontic treatment outcomes and one of the major areas researched includes the effect of orthognathic surgery on facial appearance and age perception [36, 38]. The algorithms used in these studies concluded that most patient’s appearance improved with treatment (66.4%), resulting in a younger appearance of nearly 1 year, especially after a profile altering surgery. Similar improvement was noted on facial attractiveness in 74.7% of patients, especially after lower jaw surge and the authors concluded that AI might be considered to score facial attractiveness and apparent age in orthognathic patients [38].

With regard to the assessment of growth and development and/or evaluation of growth patterns. Spampinato et al. [39] proposed and tested several deep learning approaches to assess skeletal bone age automatically in what was one of the first studies for an automated skeletal bone age assessment, tested on a public dataset and for all age ranges, races, and genders and with a source code available. Results showed an average discrepancy of 0.8 years between manual and automatic assessment and considered to be a state-of-art performance reliability as per the authors. A recent study [20] compared seven artificial intelligence algorithms—k-nearest neighbors, Naïve Bayes, decision tree, artificial neural networks, support vector machines, random forest, and logistic regression algorithms to determine the preferred method of cervical vertebrae maturation and concluded that ANN was the most stable and preferred method of determining the same.

There have been many attempts to aid orthodontists in classifying patient growth patterns [83–85]. One of the first methods of using ANN in evaluating growth occurred in 1998 where the growth of 43 untreated children was classified based on size and shape changes [30]. Nino-Sandoval et al. utilized a support vector machine to classify skeletal patterns through craniomaxillary variables but achieved only 74.51 % accuracy in the correct distinction of class II skeletal pattern from class III pattern and vice-versa [24, 44].

Applications of AI and ML that could not be described under the above four major domains were grouped under the miscellaneous category in this review and these include automated tooth segmentation either from CBCT images or dental models [33, 43], detection of activation pattern of tongue musculature [50] and evaluation of effects of a different curing unit and light-tips on temperature increase during orthodontic bonding [26].

### Limitations

Some AI applications may have been missed out due to the inclusion criteria, utilized search terms, if they were published in a language other than English and/or due to the non-inclusion in PubMed.

Due to its nature, scoping reviews are not expected to utilize a risk of bias tool to assess methodological strengths of included studies. The overall idea is to explore in a superficial way what is currently known in a specific area.

## Conclusion

- This scoping review showcases that there has been an exponential increase in the number of orthodontic studies involving various applications of AI and ML over the past three decades.
- The majority of these studies originated from the USA, followed by South Korea and China.
- The number of studies published in non-orthodontic journals (36) was found to be more extensive than those published in orthodontic specialty journals (26).
- Artificial neural networks (ANNs) were found to be the most commonly utilized AI/ML algorithm, followed by convolutional neural networks (CNNs) and support vector machine (SVM).
- The most commonly utilized AI domains were for diagnosis and treatment planning (33 studies), automated anatomic landmark detection and/or analyses (19), assessment of growth and development (4), and evaluation of treatment outcome (2).

## Supplementary Information

**Additional file 1: Supplementary table 1.** Master chart of studies included in the scoping review.

**Additional file 2: Supplementary table 2.** Distribution of studies per decade.

**Additional file 3: Supplementary table 3.** Country of origin of the study.

**Additional file 4: Supplementary table 4.** Studies published in orthodontic specialty journals or non-orthodontic journals.

## Data Availability

The datasets used and/or analyzed during the current study are available from the corresponding author on reasonable request.

## Abbreviations

IT: Information technology
AI: Artificial intelligence
ML: Machine learning
DL: Deep learning

## References

[1] Asiri SN, Tadlock LP, Schneiderman E, Buschang PH (2020). Applications of artificial intelligence and machine learning in orthodontics. APOS Trends Orthod.

[2] Haugeland J (1985). Artificial intelligence: the very idea.

[3] Morris CG (1996). Academic Press Dictionary of Science Technology.

[4] Luger GF (2005). Artificial intelligence: structures and strategies for complex problem solving.

[5] McCulloch WS, Pitts W (1943). A logical calculus of the ideas immanent in nervous activity. Bull Math Biophys..

[6] Negnevitsky M (2005). Artificial intelligence: a guide to intelligent systems.

[7] Faber J, Faber C, Faber P (2019). Artificial intelligence in orthodontics. APOS Trends Orthod.

[8] Could Artificial Intelligence Transform Healthcare? Morgan Stanley. Available from: https://www.morganstanley.com/ideas/medtech-artificial-intelligence. Accessed 12 Dec 2020.

[9] Michael W, Haugeland J (1987). Artificial intelligence: the very idea. Technol Culture..

[10] Schwartz WB, Patil RS, Szolovits P (1987). Artificial intelligence in medicine*.* Where do we stand?. N Engl J Med..

[11] Bishop CM (2006). Pattern recognition and machine learning.

[12] Ko C, Tanikawa C, Wu T, Pastewait Matthew, Jackson C B, Kwon JJ, Lee Y, Lian C, Wang Li, Shen D Machine learning in orthodontics: application review. Embracing novel technologies in dentistry and orthodontics- Forty-sixth Annual Moyers Symposium and the Forty-fourth Annual International Conference on Craniofacial Research March 1-3, 2019, Ann Arbor, Michigan.

[13] Sumathi S, Sivanandam S (2006). Introduction to data mining principles. Introduction to Data Mining and its Applications. Studies in Computational Intelligence.

[14] Mitchell TM (1999). Machine learning and data mining. Commun ACM..

[15] Breiman L (2001). Statistical modeling: the two cultures (with comments and a rejoinder by the author). Stat Sci..

[16] Vaid N (2019). Scoping studies: should there be more in orthodontic literature?. APOS Trends Orthod.

[17] Arksey H, O'Malley L (2005). Scoping studies: towards a methodological framework. Int J Soc Res Methodol..

[18] Suhail Y, Upadhyay M, Chhibber A, Kshitiz (2020). Machine learning for the diagnosis of orthodontic extractions: a computational analysis using ensemble learning. Bioengineering.

[19] Muraev AA, Tsai P, Kibardin I, Oborotistov N, Shirayeva T, Ivanov S, Ivanov S, Guseynov N, Aleshina O, Bosykh Y, Safyanova E, Andreischev A, Rudoman S, Dolgalev A, Matyuta M, Karagodsky V, Tuturov N (2020). Frontal cephalometric landmarking: humans vs artificial neural networks. Int J Comput Dent..

[20] Kök H, Acilar AM, İzgi MS (2019). Usage and comparison of artificial intelligence algorithms for determination of growth and development by cervical vertebrae stages in orthodontics. Prog Orthod..

[21] Choi HI, Jung SK, Baek SH, Lim WH, Ahn SJ, Yang IH, Kim TW (2019). Artificial intelligent model with neural network machine learning for the diagnosis of orthognathic surgery. J Craniofac Surg.

[22] Shoukri B, Prieto JC, Ruellas A, Yatabe M, Sugai J, Styner M, Zhu H, Huang C, Paniagua B, Aronovich S, Ashman L, Benavides E, de Dumast P, Ribera NT, Mirabel C, Michoud L, Allohaibi Z, Ioshida M, Bittencourt L, Fattori L, Gomes LR, Cevidanes L (2019). Minimally invasive approach for diagnosing TMJ osteoarthritis. J Dent Res..

[23] Li P, Kong D, Tang T, Su D, Yang P, Wang H, Zhao Z, Liu Y (2019). Orthodontic treatment planning based on artificial neural networks. Sci Rep..

[24] Niño-Sandoval TC, Guevara Pérez SV, González FA, Jaque RA, Infante-Contreras C (2017). Use of automated learning techniques for predicting mandibular morphology in skeletal class I, II and III. Forensic Sci Int.

[25] Jung SK, Kim TW (2016). New approach for the diagnosis of extractions with neural network machine learning. Am J Orthod Dentofacial Orthop..

[26] Aksakalli S, Demir A, Selek M, Tasdemir S (2014). Temperature increase during orthodontic bonding with different curing units using an infrared camera. Acta Odontol Scand..

[27] Mario MC, Abe JM, Ortega NR, Del Santo M (2010). Paraconsistent artificial neural network as auxiliary in cephalometric diagnosis. Artif Organs..

[28] Xie X, Wang L, Wang A (2010). Artificial neural network modeling for deciding if extractions are necessary prior to orthodontic treatment. Angle Orthod..

[29] Akdenur B, Okkesum S, Kara S, Günes S (2009). Correlation- and covariance-supported normalization method for estimating orthodontic trainer treatment for clenching activity. Proc Inst Mech Eng H..

[30] Lux CJ, Stellzig A, Volz D, Jäger W, Richardson A, Komposch G (1998). A neural network approach to the analysis and classification of human craniofacial growth. Growth Dev Aging..

[31] Ma Q, Kobayashi E, Fan B, Nakagawa K, Sakuma I, Masamune K, Suenaga H (2020). Automatic 3D landmarking model using patch-based deep neural networks for CT image of oral and maxillofacial surgery. Int J Med Robot..

[32] Yu HJ, Cho SR, Kim MJ, Kim WH, Kim JW, Choi J (2020). Automated skeletal classification with lateral cephalometry based on artificial intelligence. J Dent Res..

[33] Chung M, Lee M, Hong J, Park S, Lee J, Lee J, Yang IH, Lee J, Shin YG (2020). Pose-aware instance segmentation framework from cone beam CT images for tooth segmentation. Comput Biol Med..

[34] Lee KS, Jung SK, Ryu JJ, Shin SW, Choi J (2020). Evaluation of transfer learning with deep convolutional neural networks for screening osteoporosis in dental panoramic radiographs. J Clin Med..

[35] Kunz F, Stellzig-Eisenhauer A, Zeman F, Boldt J. Artificial intelligence in orthodontics: evaluation of a fully automated cephalometric analysis using a customized convolutional neural network. J Orofac Orthop. 2020;81(1):52-68. English. doi: 10.1007/s00056-019-00203-8. Epub 2019 Dec 18. 10.1007/s00056-019-00203-8 31853586

[36] Patcas R, Timofte R, Volokitin A, Agustsson E, Eliades T, Eichenberger M, Bornstein MM (2019). Facial attractiveness of cleft patients: a direct comparison between artificial-intelligence-based scoring and conventional rater groups. Eur J Orthod..

[37] Nishimoto S, Sotsuka Y, Kawai K, Ishise H, Kakibuchi M (2019). Personal computer-based cephalometric landmark detection with deep learning, using cephalograms on the internet. J Craniofac Surg..

[38] Patcas R, Bernini DAJ, Volokitin A, Agustsson E, Rothe R, Timofte R (2019). Applying artificial intelligence to assess the impact of orthognathic treatment on facial attractiveness and estimated age. Int J Oral Maxillofac Surg..

[39] Spampinato C, Palazzo S, Giordano D, Aldinucci M, Leonardi R (2017). Deep learning for automated skeletal bone age assessment in X-ray images. Med Image Anal..

[40] Yeom SH, Na JS, Jung HD, Cho HJ, Choi YJ, Lee JS (2019). Computational analysis of airflow dynamics for predicting collapsible sites in the upper airways: machine learning approach. J Appl Physiol (1985).

[41] Hwang JJ, Lee JH, Han SS, Kim YH, Jeong HG, Choi YJ, Park W (2017). Strut analysis for osteoporosis detection model using dental panoramic radiography. Dentomaxillofac Radiol..

[42] Wang X, Cai B, Cao Y, Zhou C, Yang L, Liu R, Long X, Wang W, Gao D, Bao B (2016). Objective method for evaluating orthodontic treatment from the lay perspective: an eye-tracking study. Am J Orthod Dentofacial Orthop..

[43] Mortaheb P, Rezaeian M (2016). Metal artifact reduction and segmentation of dental computerized tomography images using least square support vector machine and mean shift algorithm. J Med Signals Sens..

[44] Niño-Sandoval TC, Guevara Perez SV, González FA, Jaque RA, Infante-Contreras C. An automatic method for skeletal patterns classification using craniomaxillary variables on a Colombian population. Forensic Sci Int. 2016261:159.e1-159.e6. doi: 10.1016/j.forsciint.2015.12.025. Epub 2015 Dec 25. 10.1016/j.forsciint.2015.12.025 26782070

[45] Yu X, Liu B, Pei Y, Xu T (2014). Evaluation of facial attractiveness for patients with malocclusion: a machine-learning technique employing Procrustes. Angle Orthod..

[46] Kim BM, Kang BY, Kim HG, Baek SH (2009). Prognosis prediction for class III malocclusion treatment by feature wrapping method. Angle Orthod..

[47] Bianchi J, de Oliveira Ruellas AC, Gonçalves JR, Paniagua B, Prieto JC, Styner M, Li T, Zhu H, Sugai J, Giannobile W, Benavides E, Soki F, Yatabe M, Ashman L, Walker D, Soroushmehr R, Najarian K, Cevidanes LHS (2020). Osteoarthritis of the temporomandibular joint can be diagnosed earlier using biomarkers and machine learning. Sci Rep..

[48] Zhang SJ, Meng P, Zhang J, Jia P, Lin J, Wang X, Chen F, Wei X (2018). Machine learning models for genetic risk assessment of infants with non-syndromic orofacial cleft. Genomics Proteomics Bioinformatics..

[49] Sorihashi Y, Stephens CD, Takada K (2000). An inference modeling of human visual judgment of sagittal jaw-base relationships based on cephalometry: part II. Am J Orthod Dentofacial Orthop..

[50] Tolpadi AA, Stone ML, Carass A, Prince JL, Gomez AD (2018). Inverse biomechanical modeling of the tongue via machine learning and synthetic training data. Proc SPIE Int Soc Opt Eng..

[51] Laurenziello M, Montaruli G, Gallo C, Tepedino M, Guida L, Perillo L, Troiano G, Lo Muzio L, Ciavarella D (2017). Determinants of maxillary canine impaction: retrospective clinical and radiographic study. J Clin Exp Dent..

[52] Allareddy V, Wang T, Rampa S, Caplin J, Nalliah R, Badheka A, Allareddy V (2019). Prevalence and predictors of C. difficile infections in hospitalized patients with major surgical procedures in the USA: analysis using traditional and machine learning methods. Am J Surg..

[53] Thanathornwong B (2018). Bayesian-based decision support system for assessing the needs for orthodontic treatment. Healthc Inform Res..

[54] Nieri M, Crescini A, Rotundo R, Baccetti T, Cortellini P, Pini Prato GP (2010). Factors affecting the clinical approach to impacted maxillary canines: a Bayesian network analysis. Am J Orthod Dentofacial Orthop..

[55] Stephens C, Mackin N (1998). The validation of an orthodontic expert system rule-base for fixed appliance treatment planning. Eur J Orthod..

[56] Stephens CD, Mackin N, Sims-Williams JH (1996). The development and validation of an orthodontic expert system. Br J Orthod..

[57] Brown ID, Adams SR, Stephens CD, Erritt SJ, Sims-Williams JH (1991). The initial use of a computer-controlled expert system in the treatment planning of class II division 1 malocclusion. Br J Orthod..

[58] Montúfar J, Romero M, Scougall-Vilchis RJ (2018). Hybrid approach for automatic cephalometric landmark annotation on cone-beam computed tomography volumes. Am J Orthod Dentofacial Orthop..

[59] Montúfar J, Romero M, Scougall-Vilchis RJ (2018). Automatic 3-dimensional cephalometric landmarking based on active shape models in related projections. Am J Orthod Dentofacial Orthop..

[60] Gupta A, Kharbanda OP, Sardana V, Balachandran R, Sardana HK (2016). Accuracy of 3D cephalometric measurements based on an automatic knowledge-based landmark detection algorithm. Int J Comput Assist Radiol Surg..

[61] Gupta A, Kharbanda OP, Sardana V, Balachandran R, Sardana HK (2015). A knowledge-based algorithm for automatic detection of cephalometric landmarks on CBCT images. Int J Comput Assist Radiol Surg..

[62] Auconi P, Scazzocchio M, Cozza P, McNamara JA, Franchi L (2015). Prediction of class III treatment outcomes through orthodontic data mining. Eur J Orthod..

[63] Akçam MO, Takada K (2002). Fuzzy modelling for selecting headgear types. Eur J Orthod..

[64] Vucinić P, Trpovski Z, Sćepan I (2010). Automatic landmarking of cephalograms using active appearance models. Eur J Orthod..

[65] Rueda S, Alcañiz M (2006). An approach for the automatic cephalometric landmark detection using mathematical morphology and active appearance models. Med Image Comput Comput Assist Interv..

[66] Ribera NT, de Dumast P, Yatabe M, Ruellas A, Ioshida M, Paniagua B, Styner M, Gonçalves JR, Bianchi J, Cevidanes L, Prieto JC (2019). Shape variation analyzer: a classifier for temporomandibular joint damaged by osteoarthritis. Proc SPIE Int Soc Opt Eng..

[67] de Dumast P, Mirabel C, Cevidanes L, Ruellas A, Yatabe M, Ioshida M, Ribera NT, Michoud L, Gomes L, Huang C, Zhu H, Muniz L, Shoukri B, Paniagua B, Styner M, Pieper S, Budin F, Vimort JB, Pascal L, Prieto JC (2018). A web-based system for neural network based classification in temporomandibular joint osteoarthritis. Comput Med Imaging Graph..

[68] Takada K, Yagi M, Horiguchi E (2009). Computational formulation of orthodontic tooth-extraction decisions. Part I: to extract or not to extract. Angle Orthod..

[69] Grau V, Alcañiz M, Juan MC, Monserrat C, Knoll C (2001). Automatic localization of cephalometric Landmarks. J Biomed Inform..

[70] Kim H, Shim E, Park J, Kim YJ, Lee U, Kim Y (2020). Web-based fully automated cephalometric analysis by deep learning. Comput Methods Programs Biomed..

[71] Chen S, Wang L, Li G, Wu TH, Diachina S, Tejera B, Kwon JJ, Lin FC, Lee YT, Xu T, Shen D, Ko CC (2020). Machine learning in orthodontics: introducing a 3D auto-segmentation and auto-landmark finder of CBCT images to assess maxillary constriction in unilateral impacted canine patients. Angle Orthod..

[72] Skotko BG, Macklin EA, Muselli M, Voelz L, McDonough ME, Davidson E, Allareddy V, Jayaratne YS, Bruun R, Ching N, Weintraub G, Gozal D, Rosen D (2017). A predictive model for obstructive sleep apnea and Down syndrome. Am J Med Genet A..

[73] Auconi P, Caldarelli G, Scala A, Ierardo G, Polimeni A (2011). A network approach to orthodontic diagnosis. Orthod Craniofac Res..

[74] Tanikawa C, Yagi M, Takada K (2009). Automated cephalometry: system performance reliability using landmark-dependent criteria. Angle Orthod..

[75] Rudolph DJ, Sinclair PM, Coggins JM (1998). Automatic computerized radiographic identification of cephalometric landmarks. Am J Orthod Dentofacial Orthop..

[76] Ed-Dhahraouy M, Riri H, Ezzahmouly M, Bourzgui F, El Moutaoukkil A (2018). A new methodology for automatic detection of reference points in 3D cephalometry: a pilot study. Int Orthod..

[77] Neelapu BC, Kharbanda OP, Sardana V, Gupta A, Vasamsetti S, Balachandran R, Sardana HK (2018). Automatic localization of three-dimensional cephalometric landmarks on CBCT images by extracting symmetry features of the skull. Dentomaxillofac Radiol..

[78] Tanikawa C, Yamamoto T, Yagi M, Takada K (2010). Automatic recognition of anatomic features on cephalograms of preadolescent children. Angle Orthod..

[79] Hammond RM, Freer TJ (1997). Application of a case-based expert system to orthodontic diagnosis and treatment planning. Aust Orthod J..

[80] Gandedkar NH, Vaid NR, Darendeliler MA, Premjani P, Ferguson DJ (2019). The last decade in orthodontics: a scoping review of the hits, misses and the near misses! Semin in Orthod. Vol.

[81] Vaid NR, Hansa I, Bichu Y (2020). Smartphone applications used in orthodontics: a scoping review of scholarly literature. J World Fed Orthod..

[82] Lévy-Mandel AD, Venetsanopoulos AN, Tsotsos JK (1986). Knowledge-based landmarking of cephalograms. Comput Biomed Res..

[83] Brodie AG (1941). Behavior of normal and abnormal facial growth patterns. Am J Orthod Dent Orthop..

[84] Broadbent BH (1937). The face of the normal child. Angle Orthod..

[85] Ricketts RM (1972). A principle of arcial growth of the mandible. Angle Orthod..

