# Supplementary material for: Applications of artificial intelligence and machine learning in orthodontics: a scoping review
Source: Prog Orthod. 2021 Jul 5;22:18. doi: 10.1186/s40510-021-00361-9 (PMC8255249; doi:10.1186/s40510-021-00361-9)
Supplement: Supplementary file 1 — Additional file 1: Supplementary table 1. Master chart of studies included in the scoping review. [file 40510_2021_361_MOESM1_ESM.docx]

| Supplementary table 1: Master chart of studies included in the scoping review | | | | | | | | |
| --- | --- | --- | --- | --- | --- | --- | --- | --- |
| Author / Year | Country of Origin | Published in Ortho Journal | Participants | Intervention | Comparison | Outcome | AI algorithm employed | Outcome  Domain |
| Suhail Y ^18^  2020 | USA | N | 287 orthodontic pre-treatment records | Multiple machine learning models | Opinions of five orthodontic experts | Extraction versus non-extraction decision | Random forest, neural network, logistic regression | Diagnosis and treatment planning: orthodontic extraction decision |
| Muraev A ^19^  2020 | Russia | N | 330 frontal cephalograms: 300 for training artificial neural networks (ANNs) and 30 for research | Two ANNs | Three groups of doctors: expert, regular, inexperienced | Accuracy of placing cephalometric points on frontal cephalograms | ANN | Automated anatomic landmark detection and/or analysis |
| Bianchi J ^20^  2020 | USA  Brazil | N | Fifty-two clinical, biological and radiomic markers from 92 subjects | TMJ OsteoarthrItis  (TMJ-OA) | Age and sex-matched controls | Accurate diagnosis of TMJ-OA status | Logistic Regression, random forest, lightGBM, XGBoost | Diagnosis and treatment planning: detection of TMJ-OA |
| Kim H ^21^  2020 | South Korea | N | 23 landmarks from 2075 lateral cephalograms | Two-stage automated algorithm,  development of web-based application based on proposed algorithm | Manual landmarking and analysis | Accuracy of cephalometric landmark detection and automated cephalometric analysis | Stacked hourglass deep learning network | Automated anatomic landmark detection and/or analysis |
| Ma Q ^22^  2020 | Japan | N | CT images of 66 patients treated with oral and maxillofacial surgery | Patch-based deep neural network model with three-layer convolutional neural network (CNN) | Manual landmarking | Accuracy and average processing time for anatomic landmarking | CNN | Automated anatomic landmark detection and/or analysis |
| Yu H ^23^  2020 | South Korea | N | 5890 lateral cephalograms and demographic data | Transfer learning and data augmentation techniques | Manual landmarking | Automated  skeletal orthodontic diagnosis and classification | Multi-modal convolutional neural network (CNN) | Automated anatomic landmark detection and/or analysis |
| Chung M ^24^  2020 | South Korea | N | Cone beam CT images | Neural networks | Other state-of-art algorithms | Pose-aware volume of interest (VOI) realignment followed by robust tooth detection and a metal-robust framework for accurate tooth segmentation | Regression neural networks  Convolutional Neural Networks | Miscellaneous: tooth segmentation from CBCT images |
| Lee K ^25^  2020 | South Korea | N | Dataset- 680 images from patients who underwent both skeletal bone mineral density and digital panoramic radiographic examinations | Deep convolutional neural networks (CNNs) employed with various transfer learning strategies | 4 study groups: a basic CNN model with 3 convolutional layers (CNN3), visual geometry group deep CNN model (VGG-16), transfer learning model from VGG-16 (16_TF) and fine-tuning with transfer learning model (VGG-16_TF_FT) | Classification of specific features of osteoporosis in dental panoramic radiographs | CNNs | Diagnosis and treatment planning: screening of osteoporosis |
| Kunz F ^26^  2020 | Germany | Y | 18 landmarks on 1972 cephalometric x-rays as training set and 12 landmarks on 50 x-rays as research set | Specialized AI algorithm | 12 experienced examiners | Accuracy of cephalometric landmark detection and automated cephalometric analysis | Convolutional neural network | Automated anatomic landmark detection and/or analysis |
| Kok H ^27^  2019 | Turkey | Y | 19 reference points on 2^nd^,3^rd^ and 4^th^ cervical vertebrae, 20 different linear measurements on  cephalometric radiographs of 300 patients aged between 8 and 17 years | Seven frequently used AI classifiers | Performance of these seven algorithms with each other | Determination of growth and development by cervical vertebrae stages | k-Nearest Neighbors, Naïve Bayes, Decision tree, Artificial Neural Networks, Support Vector Machines, Random Forest and Logistic Regression | Assessment of growth and development |
| Choi H ^28^  2019 | South Korea | N | 316 patients- 160 planned for surgical treatment and 156 for non-surgical;  12 measurement values from lateral cephalogram and 6 additional indices | New artificial intelligence algorithm | Actual clinical diagnosis | Surgery/non-surgery decision and extraction determination | 2-layer artificial neural network (ANN) with one hidden layer | Diagnosis and treatment planning: orthognathic surgery and orthodontic extraction decision |
| Shoukri B ^29^  2019 | USA | N | 17 TMJ Osteoarthritis patients (TMJ-OA) with signs and symptoms of disease for less than 10 years and average age 39.9+/-11.7 years | Artificial neural network trained on 259 other condyles | Clinical expert’s classification | Protein expression levels, clinical symptoms and condylar surface morphology for diagnosis of TMJ Osteoarthritis | Neural network | Diagnosis and treatment planning: detection of TMJ-OA |
| Patcas R ^30^  2019 | Switzerland  China | Y | Frontal and profile images of 20 treated, left-sided cleft patients and 10 controls | Convolutional Neural Networks (CNNs) trained on >17 million ratings for attractiveness | Assessments of 15 laypeople, 14 orthodontists and 10 oral surgeons performed on a visual analogue scale | Facial attractiveness of treated cleft patients and controls | CNNs | Evaluation of treatment outcome |
| Chen S ^31^  2019 | China  USA | Y | CBCT images of 30 patients with unilaterally impacted canines | Machine learning (ML) algorithm to quantify volumetric skeletal maxillary discrepancies | 30 healthy controls | 3D auto-segmentation and auto-landmark finder of CBCT images to assess maxillary constriction in unilateral impacted canine patients | ML utilizing Learning-based multi-source IntegratioN frameworK for segmentation (LINKS) | Diagnosis and treatment planning: maxillary constriction and unilateral impacted canines |
| Yeom S ^32^  2019 | South Korea | N | 4 major geometric parameters on 160 cases | Predictive model using Gaussian process regression | CT data of patients with normal geometry | Airflow dynamics for predicting collapsible sites in upper airways and identify degree of OSA symptoms in patients as normal-mild, moderate and severe | Gaussian process regression  Support Vector Machine | Diagnosis and treatment planning: airflow dynamics, upper airway collapsible sites and Obstructive Sleep Apnea |
| Ribera N ^33^  2019 | USA  Brazil | N | 259 TMJ CBCT scans for the training set and 34 for the testing dataset | Deep learning neural network | 8 other supervised ML algorithms | Classifier for disease staging of pathological bony changes in TMJ-OA | Shape Variation Analyzer- SVA | Diagnosis and treatment planning: detection of TMJ-OA |
| Li P ^34^  2019 | China | N | 302 orthodontically treated cases | Artificial Neural Network (ANN) | k-Nearest Neighbors (k-NN) | Predict orthodontic treatment plans, including determination of extraction/ non-extraction decision, extraction patterns and anchorage patterns | Three-layer fully connected Multilayer perceptron artificial neural networks (ANNs) | Diagnosis and treatment planning: orthodontic extraction decision |
| Nishimoto S ^35^  2019 | Japan | N | 10 skeletal cephalometric landmarks plotted on 219 lateral cephalogram images gathered through internet- randomly divided into 153 training images and 66 testing images | Deep learning neural network built for regression analysis of cephalometric landmarks’ coordinate values | Manually plotted points | Accuracy of personal computer-based cephalometric landmark detection and analysis | Convolutional neural network | Automated anatomic landmark detection and/or analysis |
| Allareddy V ^36^  2018 | USA | N | Nationwide Inpatient Sample from 2012 to 2014-19931347 hospitalized patients who underwent a major surgical procedure  Half aged between 40-64 and half above 65 years  52.6% females | Machine learning approach | Traditional approach | Prevalence and predictors of occurrence of Clostridium difficile infections (CDI) in hospitalized patients | Bayesian Rule Set model | Diagnosis and treatment planning: occurrence of CDI |
| Zhang S ^37^  2018 | China | N | Blood samples from Han and Uyghur infants with Non-Syndromic Cleft Lip/Palate (NSCL/P) | 43 candidate SNPs- Single Nucleotide Polymorphisms previously detected using GWAs- Genome-wide association | Age and sex-matched controls | Predictors of genetic risk assessment for occurrence of NSCL/P | Logistic regression | Diagnosis and treatment planning: genetic risk assessment of NSCL/P |
| Patcas R ^38^  2018 | Switzerland  Germany | N | 2164 pre-treatment photographs of 146 consecutive orthognathic patients | Orthognathic surgery | Post-treatment photographs | Impact of orthognathic surgery on facial attractiveness and estimated age | Convolutional Neural Networks | Evaluation of treatment outcome |
| Montufar J ^39^  2018 | Mexico | Y | 18 landmarks located on 24 CBCT head scans from public dataset | Hybrid algorithm | Manual landmarking | Automatic cephalometric landmark annotation on cone-beam computed tomography volumes | Active shape models | Automated anatomic landmark detection and/or analysis |
| Ed-Dhahraouy M ^40^  2018 | Morocco | Y | 21 reference points on craniofacial structures from 5 CBCT images | Automatic algorithm | Manual landmarking | Automatic identification of reference points in 3D cephalometry | Specific application designed in C++ language | Automated anatomic landmark detection and/or analysis |
| Dumast P ^41^  2018 | USA | N | Clinical, biological and imaging markers for 34 subjects- 17 with TMJ-Osteoarthritis (TMJ-OA) and 17 age and sex matched control subjects (39.4 ± 15.4 years), without any sign or symptom of TMJ-OA | Deep neural network | Clinician consensus | Remotely computed, web-based system for neural network-based classification in TMJ-OA | Shape Variation Analyzer (SVA), and flexible web-based system for data storage, computation and integration (DSCI) | Diagnosis and treatment planning: classification of TMJ-OA |
| Montufar J ^42^  2018 | Mexico | Y | 18 tridimensional landmarks located on 24 random CBCT scans from public dataset | Automatic detection algorithm | Manual landmarking | Automatic cephalometric landmark localization on 3-D CBCT volumes | 2 trained active shape models | Automated anatomic landmark detection and/or analysis |
| Tolpadi A ^43^  2018 | USA | N | 262 contractile elements of tongue musculature | Machine learning algorithm | Semi-manual approach | Estimation of activation patterns and resulting deformations of tongue musculature | 30 decision trees using random forest methodology | Miscellaneous:  estimation of activation patterns of tongue musculature |
| Neelapu B ^44^  2018 | India | N | 20 landmarks overall, 12 on mid-sagittal plane | Automatic detection algorithm | Manual landmarking | Accuracy of localization of 3D cephalometric landmarks on CBCT useful for both cephalometric and upper airway volumetric analysis | Unspecified | Automated anatomic landmark detection and/or analysis |
| Thanathornwong B ^45^  2018 | Thailand | N | One thousand permanent dentition patient data sets from hospital record system | Clinical decision support system | Judgements of two orthodontists | Help general practitioners assess need for orthodontic treatment | Bayesian network (BN) | Diagnosis and treatment planning: assessment of need for orthodontic treatment |
| Laurenziello M ^46^  2017 | Italy | N | Clinical and radiographic analysis on 109 patients aged between 9 and 10 years at time of first evaluation,  two years follow up period | Machine learning methods to build a predictive model | Univariate and multivariate statistical analyses, including correlation among studied variables and principal components analysis | Evaluate determinants of maxillary canine impaction taking into account both canine position related variables and pattern of facial growth | Random forest | Diagnosis and treatment planning: maxillary canine impaction |
| Nino-Sandoval T ^47^  2017 | Colombia | N | 229 standardized lateral radiographs of Colombian young adults of both sexes between ages 18-25 | Automated learning techniques | Actual measurements | Predict mandibular morphology through craniomaxillary variables in skeletal class I, II and III patterns | Artificial Neural Networks and Support Vector Regression | Diagnosis and treatment planning-  classification of skeletal patterns |
| Hwang J ^48^  2017 | South Korea | N | 227 randomly selected panoramic radiographs from 2012-2015 | Osteoporosis | Non-osteoporotic patient groups | Development of an osteoporosis detection model based on panoramic radiography | Decision tree and Support Vector Machine | Diagnosis and treatment planning: osteoporosis detection |
| Skotko B ^49^  2017 | USA  Italy | N | 130 patients of Down syndrome aged 3-24 years | Mild Obstructive Sleep Apnea (OSA) | Moderate or severe sleep OSA | Predict which patients with DS were unlikely to have moderate to severe OSA and may not need a diagnostic sleep study | Logic Learning Machine | Diagnosis and treatment planning: association between OSA and Down syndrome |
| Spampinato C ^50^  2016 | Italy | N | Public X-ray dataset containing 1391 left-hand scans of children up to 18 years age, divided by gender and race | Deep learning method | Two bone age values provided by two expert radiologists using Greulich and Pyle and Tanner-Whitehouse method | Automated skeletal bone age assessment from hand/wrist X-ray images | Convolutional Neural Networks  Regression network | Assessment of growth and development |
| Wang X ^51^  2016 | China | Y | Scanpaths of 88 laypersons observing repose and smiling photographs of normal subjects and pre-treatment and post-treatment malocclusion patients | Machine learning method | Aesthetic component of IOTN | Objectively quantify effect of malocclusion on facial perception and impact of orthodontic treatment on malocclusion from lay perspective | Linear regression and Support Vector Machine | Diagnosis and treatment planning: assessment of orthodontic treatment needs and quantify orthodontic treatment outcomes |
| Mortaheb P ^52^ 2016 | Iran | N | 14 cone-beam CT (CBCT) images | Multi-step auto-segmentation method | Conventional methods | Automatic segmentation and metal artefact reduction of dental CBCT images | Support Vector Machine and Mean Shift Algorithm | Miscellaneous: tooth segmentation from CBCT images |
| Jung S ^53^  2016 | South  Korea | Y | 156 subjects- 96 learning set, 60 data set,  12 cephalometric variables and additional 6 indexes | Artificial intelligence expert system | Actual clinical diagnosis | Diagnosis of extraction versus non-extraction and detailed diagnosis of extraction patterns | Two-layer neural network | Diagnosis and treatment planning: orthodontic extraction decision |
| Gupta A ^54^  2015 | India | N | 51 cephalometric measurements- 28 linear, 16 angles and 7 ratios on 30 CBCT images based on 21 cephalometric landmarks | Automatic cephalometric analysis | Manual analysis by 3 orthodontists | Accuracy of 3D cephalometric measurements | Automatic detection  algorithm | Automated anatomic landmark detection and/or analysis |
| Nino-Sandoval T ^55^  2015 | Colombia | N | Lateral cephalograms of 229 Colombian young adults of both sexes | Automatic non-parametric method for classification of skeletal patterns using craniomaxillary variables | Classification based on manual methods | Accuracy, precision and recall of skeletal classes | SVM- Support Vector Machine | Diagnosis and treatment planning-  classification of skeletal patterns |
| Gupta A ^56^  2015 | India | N | 20 cephalometric landmarks on 30 CBCT images | Knowledge-based algorithm developed in MATLAB programming environment | Manual marking by 3 orthodontists | Accuracy of automatic 3D cephalometric landmark detection | Automatic detection  algorithm | Automated anatomic landmark detection and/or analysis |
| Auconi P ^57^  2015 | Italy  USA | Y | Cephalometric data of 54 class III patients (32 females, 22 males) taken before (T1, mean age 8.2 +/- 1.6 years) and after (T2, mean age 14.6 +/- 1.8 years) early rapid maxillary expansion and facemask therapy followed by fixed appliances | Model derived from computational analyses | Actual clinical treatment results obtained | Prediction of class III treatment outcomes | Fuzzy clustering repartition and network analysis | Diagnosis and treatment planning: prediction of orthodontic treatment outcomes- class III malocclusion |
| Yu X ^58^  2014 | China | Y | 101 landmarks identified on smiling photographs of 168 randomly selected patients from 6 universities in China divided into 9 groups each with equal number of class I, II and III malocclusions, taken before and after orthodontic treatment | Machine-learning technique | 69 expert Chinese orthodontists | Objective method for evaluation of facial attractiveness from a set of orthodontic photographs | Procrustes superimposition  SVR- Support Vector Regression function | Diagnosis and treatment planning: evaluation of facial attractiveness |
| Aksakalli S ^59^  2013 | Turkey | N | Thermal records of fifty-two human premolar teeth bonded with brackets | Light-emitting diode (LED)  Curing distance from tooth surface | High intensity halogen  curing distances from 10 mm away from tooth surface | Temperature increase during orthodontic bonding | Artificial Neural Networks | Miscellaneous: effects of different curing unit and light-tips on temperature increase during orthodontic bonding |
| Auconi P ^60^  2011 | Italy | Y | 104 individuals with mixed dentition, age range of 7 to 13 years and with no previous orthodontic treatment | Network analysis | Actual clinical diagnosis | Detect and visualize most interconnected clinical, radiographic and functional data pertaining to characteristics of class II and class III malocclusions | Unspecified | Assessment of growth and development |
| Tanikawa C ^61^  2010 | Japan | Y | 859 lateral cephalograms divided into group P- 400 with permanent dentition and group M- 459 mixed dentition  59 cephalograms in group M used for system test and remaining films from both groups used for system development | Systems S(P), S(M) and S(P+M) determined landmarks | Established norms | Automatic recognition of dentoskeletal traits on cephalograms of pre-adolescents and examination of performance reliability | Unspecified | Automated anatomic landmark detection and/or analysis |
| Mario M ^62^  2010 | Brazil | N | 120 orthodontic patients | Paraconsistent artificial neural network (PANN) model | 3 orthodontic experts | Applied cephalometric analysis to measure skeletal and dental discrepancies and establish cephalometric diagnosis- expressed in degrees of skeletal, anteroposterior and dental discrepancy, pertinent to upper and lower incisors | Artificial neural network | Automated anatomic landmark detection and/or analysis |
| Vucinic P ^63^  2010 | Serbia | Y | 60 randomly selected, hand-annotated digital cephalograms of subjects between 7.2 to 25.6 years of age | Automated system | Unspecified | Accuracy of automatic landmarking of cephalograms | AAM- active appearance model | Automated anatomic landmark detection and/or analysis |
| Nieri M ^64^  2010 | Italy | Y | 168 patients with infraosseous impacted maxillary canines | Diagnosis of network model | Actual clinical treatment outcome | Evaluation of relative roles and possible causal relationships among demographic, orthodontic and periodontal variables affecting diagnosis and final treatment outcome of impacted maxillary canines | Bayesian network analysis | Diagnosis and treatment planning: prediction of orthodontic treatment outcomes- impacted maxillary canines |
| Xie X ^65^  2010 | China | Y | 200 subjects aged between 11 to 15 years - 120 extraction treatment and 80 non-extraction treatment  Data from 180 patients constituted training set and other 20 used as testing set | Decision-making expert system (ES) | Actual clinical diagnosis and treatment | Decide need for extractions for orthodontic treatment and uncover factors that affect extraction decision making process | Back Propagation (BP) Artificial Neural Network model | Diagnosis and treatment planning: orthodontic extraction decision |
| Akdenur B ^66^  2009 | Turkey | N | EMG signals from right anterior temporal muscles, recorded from 20 children aged 8-13 years with class II malocclusion undergoing orthodontic treatment | Correlation and covariance supported normalization method (CCSNM) | 4 other normalization methods: minimum-maximum normalization, z-score, decimal scaling and line-base normalization | Estimate effect of orthodontic trainer on anterior temporal muscle / clenching activity | Artificial Neural Network with Levenberg-Marquardt learning algorithm | Diagnosis and treatment planning: trainers for clenching |
| Tanikawa C ^67^  2009 | Japan | Y | 65 lateral cephalograms | Automated system | Confidence ellipses derived from scattergrams | Evaluate reliability of system that performs automatic recognition of anatomic landmarks and adjacent structures on lateral cephalograms | Projected Principal Edge Distribution  (PPED) | Automated anatomic landmark detection and/or analysis |
| Takada K ^68^  2009 | Japan | Y | 188 conventional orthodontic records of patients with good treatment outcomes | Mathematical model | Actual clinical treatments | Simulate extraction decision in optimizing orthodontic treatment outcome and formulate morphologic traits sensitive to optimizing tooth-extraction/non-extraction decisions | Pattern-  matching technique | Diagnosis and treatment planning: orthodontic extraction decision |
| Kim B ^69^  2009 | South  Korea | Y | 38 patients (15 boys, 23 girls, mean age 8.53 +/- 1.36 years), diagnosed with class III malocclusion, received 1^st^ phase- orthopedic and 2^nd^ phase- fixed orthodontic treatments, divided into 2 group, 18- poor prognosis and 20- good prognosis | Feature wrapping method (FW) | Conventional statistical methods such as Discriminant Analysis- DA | Identify which cephalometric markers show highest classification accuracy in prognosis prediction for class III malocclusion | Support vector machine (SVM) used in conjunction with FW method | Diagnosis and treatment planning: prediction of orthodontic treatment outcomes- class III malocclusion |
| Rueda S ^70^  2006 | Spain | N | 96 hand-annotated images | Automated system | Manual landmarking | Automatic cephalometric landmark detection | Active Appearance Models (AAMs) combined with mathematical morphology | Automated anatomic landmark detection and/or analysis |
| Akcam O ^71^  2002 | Turkey  Singapore | Y | 85 cases | Computer-assisted inference model | Clinical judgement of 8 experienced orthodontists | Selection of appropriate type of headgear appliance | Fuzzy modelling | Orthodontic diagnosis and treatment planning- selection of type orthodontic appliance |
| Grau V ^72^  2001 | Spain | N | 17 landmarks on 20 lateral cephalograms | Automated system | Manual landmarking | Automatic cephalometric landmark detection | Pattern detection algorithm based on mathematical morphology techniques | Automated anatomic landmark detection and/or analysis |
| Sorihashi Y ^73^  2000 | Japan | Y | Orthodontic records of 175 adult females | Automatic inference system | Clinical judgement of 7 orthodontic experts | Inference modeling of human visual judgment of sagittal base relationships based on cephalometry and development of decision-making system for orthodontic diagnosis and treatment planning | Multiple regression model | Orthodontic diagnosis and treatment planning-  assessment of sagittal skeletal discrepancy |
| Stephens C ^74^  1998 | UK | Y | Independent sample of 40 case records | Rule-based orthodontic expert system | Panel of 12 orthodontists | Appropriateness of advice designed to plan orthodontic treatment with pre-adjusted bracket appliance | Expert systems | Diagnosis and treatment planning- fixed orthodontic mechanotherapy |
| Lux C ^75^  1998 | Germany | N | Lateral cephalograms of 43 orthodontically untreated children, taken at ages of 7 to 15 | Self-organizing neural maps | Conventional cephalometric methods | Classify craniofacial growth and monitor relationships of various growth patterns | Artificial neural network  Among the 1^st^ studies to employ artificial neural networks for analysis and classification of growth-related skeletal changes | Assessment of growth and development |
| Rudolph D ^76^  1998 | USA | Y | 15 landmarks identified on a set of 14 test images | Automated system | Manual landmarking | Automatic computerized radiographic identification of cephalometric landmarks | Spatial spectroscopy (SS) | Automated anatomic landmark detection and/or analysis |
| Hammond R ^77^  1997 | Australia | Y | Case-base  300 cases  Test-set  30 consecutive cases | Computer-generated treatment plan | Actual treatment plan | Application of a case-based expert system to orthodontic diagnosis and treatment planning | Expert System | Diagnosis and treatment planning: broad-based |
| Stephens C ^78^  1996 | UK | Y | 20 cases selected from past cases  treated by dental practitioners | Recommendation from Expert system | Recommendations from orthodontic panel | Development and validation of an orthodontic expert system | Expert system | Diagnosis and treatment planning: selection of cases suitable for removable orthodontic appliances |
| Brown I ^79^  1991 | UK | Y | Treatment plans produced for 31 non-prior approval cases drawn from material provided by Dental Practice Board | Computer-based expert system | Treatments actually carried out by practitioners | Provide orthodontic advice for treatment planning for class II division 1 malocclusion | Expert system | Diagnosis and treatment planning: class II division 1 malocclusion |
