# Supplementary material for: Applications of artificial intelligence and machine learning in orthodontics: a scoping review
Source: Prog Orthod. 2021 Jul 5;22:18. doi: 10.1186/s40510-021-00361-9 (PMC8255249; doi:10.1186/s40510-021-00361-9)
Supplement: Supplementary file 2 — Additional file 2: Supplementary table 2. Distribution of studies per decade. [file 40510_2021_361_MOESM2_ESM.docx]

| Supplementary table 2: Distribution of studies per decade | | |
| --- | --- | --- |
| Decades | Number of Studies | Reference Number |
| 2011-2020 | 43 | ^18,19,20,21,22,23,24,25,26,27,28,29,30,31,32,33,34,35,36,37,38,39,40,41,42,43,44,45,46,47,48,49,50,51,52,53,54,55,56,57,58,59,60^ |
| 2001-2010 | 12 | ^61,62,63,64,65,66,67,68,69,70,71,72^ |
| 1991-2000 | 7 | ^73,74,75,76,77,78,79^ |
