# Supplementary material for: Applications of artificial intelligence and machine learning in orthodontics: a scoping review
Source: Prog Orthod. 2021 Jul 5;22:18. doi: 10.1186/s40510-021-00361-9 (PMC8255249; doi:10.1186/s40510-021-00361-9)
Supplement: Supplementary file 3 — Additional file 3: Supplementary table 3. Country of origin of the study. [file 40510_2021_361_MOESM3_ESM.docx]

| Supplementary table 3: Country of origin of the study | | |
| --- | --- | --- |
| Country of origin of the study | Number of Studies | Reference Number |
| USA | 11 | ^18,20,29,31,33,36,41,43,49,57,76^ |
| South Korea | 9 | ^21,23,24,25,28,32,48,55,69^ |
| China | 7 | ^30,31,34,37,51,58,65^ |
| Japan | 6 | ^22,35,61,67,68,73^ |
| Italy | 6 | ^46,49,50,57,60,64^ |
| Turkey | 4 | ^27,59,66,71^ |
| Brazil | 3 | ^20,33,62^ |
| India | 3 | ^44,54,56^ |
| UK | 3 | ^74,78,79^ |
| Germany | 2 | ^26,38,75^ |
| Switzerland | 2 | ^30,38^ |
| Mexico | 2 | ^39,42^ |
| Colombia | 2 | ^47,55^ |
| Spain | 2 | ^70,72^ |
| Russia | 1 | ^19^ |
| Morocco | 1 | ^40^ |
| Thailand | 1 | ^45^ |
| Iran | 1 | ^52^ |
| Serbia | 1 | ^63^ |
| Singapore | 1 | ^71^ |
| Australia | 1 | ^77^ |
