# Supplementary material for: Applications of artificial intelligence and machine learning in orthodontics: a scoping review
Source: Prog Orthod. 2021 Jul 5;22:18. doi: 10.1186/s40510-021-00361-9 (PMC8255249; doi:10.1186/s40510-021-00361-9)
Supplement: Supplementary file 4 — Additional file 4: Supplementary table 4. Studies published in orthodontic specialty journals or non-orthodontic journals. [file 40510_2021_361_MOESM4_ESM.docx]

| Supplementary table 4: Studies published in orthodontic specialty journals or non-orthodontic journals | | |
| --- | --- | --- |
| Type of publication | Number of studies | Reference Number |
| Non-orthodontic journals | 36 | ^18,19,20,21,22,23,24,25,28,29,32,33,34,35,36,37,38,41,43,44,45,46,47,48,49,50,52,54,55,56,59,62,66,70,72,75^ |
| Orthodontic journals | 26 | ^26,27,30,31,39,40,42,51,53,57,58,60,61,63,64,65,67,68,69,71,73,74,76,77,78,79^ |
